# OpenPlant: A Large-Scale Benchmark Dataset for Agricultural Plant Classification Using CNNs, ViTs, and VLMs

**DOI:** 10.3390/plants15050727

**Published:** 2026-02-27

**Authors:** Kaiqi Liu, Wei Sun, Guanping Wang, Quan Feng, Hui Li

**Affiliations:** College of Mechanical and Electrical Engineering, Gansu Agricultural University, Lanzhou 730070, China; liukq@gsau.edu.cn (K.L.); wangguanping@gsau.edu.cn (G.W.); fquan@sina.com (Q.F.); lihu@gsau.edu.cn (H.L.)

**Keywords:** precision agriculture, plant classification, agricultural datasets, deep learning, vision language models

## Abstract

Accurate plant classification based on deep learning is important for precision agriculture, such as weed control, crop monitoring, and smart farming systems. The accuracies of deep learning models rely on datasets. Although many datasets have been proposed in recent decades, they have the common limitations in terms of scale, less environmental diversity, and challenges of data integration. To solve these problems, in this paper, we introduce a new dataset named OpenPlant, which is a large-scale and open dataset containing 635,176 RGB images across 1167 plant species. OpenPlant includes diverse growth stages of plants, plant structures, and environmental conditions, and its annotations were carefully verified to ensure quality. The proposed OpenPlant can be a benchmark for agricultural plant classification. In this paper, we benchmarked 10 widely used convolutional neural networks (CNNs), 6 vision transformers (ViTs), and 12 vision–language models (VLMs) to provide a comprehensive evaluation. The OpenPlant dataset offers a comprehensive benchmark for agricultural research using deep learning and the results provide insights into future directions.

## 1. Introduction

With the rapid development of artificial intelligence (AI) and deep learning technologies, precision agriculture research is gradually moving towards data-driven approaches, much like to computer vision research. Plant classification—the task of identifying plant species from input images using deep learning—has been widely applied to support precision agriculture tasks [1,2]. In modern agricultural systems, plant classification is a fundamental and specialized task that is critical for weed management, invasive species control, and crop monitoring [3,4].

The performance of plant classification using deep learning models depends on the image datasets [5,6]. Deep learning models trained on large-scale datasets are generally more robust to variations in plant appearance. These variations are caused by differing growth conditions, growth stages, lighting environments, and camera angles. Such factors are particularly prevalent in agriculture [7,8]. However, most existing agricultural datasets are limited in both scale and diversity. This limitation makes it hard to train large deep learning models [9,10]. Common limitations of datasets used in the existing deep learning models can be summarized into four main categories. (1) Small number of classes. Most datasets contain a small number of species, often fewer than 60, as they typically focus on specific crops or diseases (as shown in Table 1), such as the AppleLeaf9 dataset [11] and the Sugarcane Disease Dataset [12]. This limits the generalizability of deep learning models across diverse species and tasks. (2) Limited environment diversity. Each dataset is focused on a specific environmental condition, such as the Open Plant Phenotype Database (OPPD) [13], PlantVillage [14], and PLD [15], which are collected under controlled laboratory conditions, and the deep learning models trained on these datasets often fail to apply in real-world field environments. (3) Challenge of data integration. Different datasets use different levels of labels, different annotations, and even some datasets are difficult to access, such as PDDD [16] and PDD271 [17]. It is hard to integrate these datasets for building a large foundation deep learning model.

These limitations highlight the need for diverse and large-scale agricultural plant image datasets to advance deep learning applications in precision farming. Despite the well-known impact of large-scale datasets such as ImageNet on computer vision, the agricultural domain still lacks a standardized resource supporting a broad range of plant classification tasks, even a decade after the release of PlantVillage, the most widely used dataset in the field. The challenge is becoming more critical as smart agriculture systems move toward comprehensive identification capabilities rather than specific tasks [18].

Another challenge is that reported performances are closely related to specific datasets. Current research on agricultural plant classification shows significant difference in model selection, from convolutional neural networks (CNNs) to vision transformers (ViTs). Some models achieve strong performance on specific datasets, but many of these datasets are not publicly available. This limitation makes it difficult to evaluate and compare model performance. Without standardized benchmarking datasets, it is challenging to determine which models can enhance agricultural vision capabilities across diverse environments and scales. In addition, foundation models trained on large datasets provide robust baseline capabilities for specific agricultural tasks. These capabilities can motivate the development of large-scale datasets to support effective transfer learning [19].

**Figure 1:**
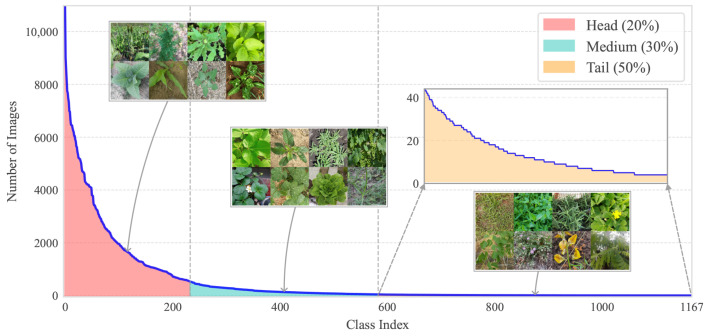
Long-tail Distribution of the OpenPlant Dataset. The distribution reveals a decline in sample frequency across different class categories. Head classes (233 species, 20%) have 536–10,981 images per class; medium classes (350 species, 30%) have 44–536 images per class; and tail classes (584 species, 50%) have 4–44 images per class. Representative images were randomly sampled from three category.

In this study, we present a new dataset named OpenPlant and evaluate it to address the various complexities and challenges in precision agriculture. We collected and organized 635,176 images of 1167 plant species. This significantly expands the category scale compared to existing datasets. To ensure annotation quality, all images were carefully checked and verified. We aim to enhance data accessibility and provide a valuable resource for future agricultural computer vision research by providing the OpenPlant dataset and alongside performance evaluations. Figure 1 exhibits its long-tail distribution. This distribution reflects the natural frequency variation in plant species. It also includes representative examples for head, medium, and tail frequency categories.

We also conducted extensive experiments on OpenPlant. We evaluated 16 widely used CNN and ViT models to establish fair performance benchmarks and guide model selection. In addition, we assessed 12 vision-language models (VLMs) to investigate their potential for plant classification within these dataset. These evaluations help to establish performance benchmarks for various algorithms in agricultural classification tasks. They also help to offer insights into model architecture suitability and reveal taxonomic factors influencing classification performance. The main contributions can be summarized as follows:

- Comprehensive review: We present a detailed survey of existing public datasets and classification methods for agricultural plant recognition. The survey includes information on image scale, annotation quality, and data sharing mechanisms. We also discuss recent developments in CNNs, ViTs, and VLMs for this task.
- OpenPlant dataset construction: We integrate 635,176 RGB images from 41 open-source repositories into a hierarchical taxonomic framework covering 1167 species. To the best of our knowledge, OpenPlant is among the largest agricultural plant datasets in both taxonomic diversity and image scale.
- Multi-dimensional performance evaluation: We perform a comprehensive benchmarking of 10 CNNs, 6 ViTs, and 12 VLMs on OpenPlant, providing a detailed analysis of performance. This evaluation framework is intended to advance research in fine-grained plant classification and broader agricultural computer vision applications.

## 2. Related Works

In this section, we comprehensively review the related methods and existing datasets.

### 2.1. Datasets in Agriculture

The widespread use of deep learning in agriculture has led to the creation of high-quality datasets. Since 2015, the number of publicly available image datasets for precision agriculture has increased. These datasets serve as a critical foundation for plant disease identification, weed detection, and crop phenotyping analysis. There is considerable variability in scale, annotation quality, and environmental diversity. This variability affects model generalizability and deployment effectiveness. We reviewed important datasets in agricultural plants used in computer vision and discussed their characteristics and limitations.

These datasets can be divided into three groups. The first group is focusing on plant disease classification. PlantVillage, a large disease recognition dataset, includes 54,309 images of 14 crop species and 38 disease and healthy classes. However, its controlled laboratory conditions have same backgrounds and lighting. These conditions may not fully capture the complexity of real-world agricultural environments. Similar limitations affect other collections, such as the Potato Disease Leaf Dataset [15] and the Corn Leaf Disease Dataset [20]. Some datasets are designed for specific crops, such as the Cassava Disease Classification Dataset [21] with 21,400 images, and Cotton Plant Disease Dataset [22] with 26,100 images, providing coverage for these important cash crops. Many datasets, such as the Rice Diseases Image Dataset [23] with 5447 images and Banana Leaf Disease Images [24] with 1288 images, primarily focus on single-leaf observations and do not capture whole-plant phenotypic information. Furthermore, existing datasets generally capture static states and overlook the dynamic development of plants. These limitations, including the relatively small size and environmental uniformity of many collections, bring challenges for developing general and robust agricultural vision systems in diverse conditions. The PlantPAD [25] attempts to address some of these limitations by including 421,314 images, 63 crops, and 310 diseases. However, we cannot get access to the dataset directly, which limits its further use.

The second group of datasets mainly focuses on weed control. This task presents challenges due to the visual similarities between crops and weeds at various growth stages. Current weed datasets show strong regional specificity. For example, DeepWeeds [26] targets Australian rangeland weeds, while CottonWeedID15 [27] focuses on weeds common in U.S. cotton production systems. This may limit model transferability across different agricultural regions and ecosystems. Additionally, the annotation methods are not consistent across datasets. Some detection-oriented datasets, like Weed25 [28] and CottonWeedDet12 [29], provide bounding box annotations. Others offer only image-level labels, which complicates dataset integration and model development for diverse weed control applications.

Another challenge is the limited study of crop–weed interactions within existing datasets. Most collections focus on weed identification, not adequately observing the competitive relationships between crops and weeds in the field. The Carrot-Weed segmentation dataset [30] attempts to address this gap but lacks diversity in crop types. The Weed Growth Stage Estimator dataset [31] contains images of 18 weed species across various environmental conditions, while its annotation only provides the growth stage. DeepSeedling [32] was developed for counting cotton and weed seedlings, which is unsuitable for crop classification. More recently, the CWD30 dataset [33] includes 219,770 images across 30 classes (10 crops and 20 weeds), demonstrating great progress. Nevertheless, it continues to face challenges, particularly in early growth stages where some images may not clearly show the target weeds or crops, complicating visual identification.

The third group of datasets includes general plant databases that offer broader taxonomic coverage but present challenges for agricultural applications. PlantNet-300K [34], with 306,146 images of 1081 species, is one of the large plant image collections available. However, variations in image quality and annotation reliability may reduce its usefulness for precision agriculture applications. These limitations indicate a gap between general databases and the requirements of agricultural vision systems.

Despite the increase in agricultural datasets, several limitations remain prevalent across them. These limitations suggest the need for a comprehensive and integrated agricultural dataset that covers more species to support deep learning models for benchmarking.

### 2.2. Computer Vision Models in Agriculture

In recent years, deep learning models have been extensively used in research focused on plant phenotyping and growth monitoring. CNNs, with their configurable depths and improved feature extraction, are highly effective in agricultural classification systems. These multi-layer frameworks help precisely identify crop disease phenotypes, differentiate varieties, and characterize growth stages. For example, models such as Inception, ResNet, and DenseNet demonstrate exceptional performance in these image classification tasks [35,36,37].

Recent advances in deep learning, particularly the application of vision transformers (ViTs), have improved the accuracy of agricultural plant image classification. ViTs use self-attention mechanisms to handle larger image contexts, enabling the model to more effectively identify plant phenotypes. Sun et al. [38] proposed an improved Transformer network that integrates a multi-scale convolution technique to effectively segment crops and weeds in sugarcane fields. This method achieved an accuracy of 96.97% on a custom dataset. Similarly, Thakur et al. [39] introduced a hybrid model combining ViT with CNN. This model merges CNN’s local feature extraction with ViT’s global attention for plant leaf disease classification, achieving an accuracy of 98.86% on the PlantVillage dataset.

Deep learning-based classification approaches have demonstrated high accuracy and performance in agricultural plants. However, existing research often focuses on specific crops within certain datasets. This can risk overfitting and result in classifiers with limited generalization capabilities. Additionally, many studies concentrate on enhancing deep learning algorithms. In such cases, custom CNN or ViT architectures may incur higher computational costs.

### 2.3. Vision Language Models in Agriculture

Recent advances in vision-language models (VLMs) have shown considerable potential for precision agricultural applications. By integrating computer vision with natural language processing capabilities, VLMs facilitate a multimodal understanding of visual-textual relationships to address complex agricultural tasks. Awais et al. [40] proposed AgroGPT, the first efficient agricultural VLM with domain-adaptive dialog capabilities. It targets specific challenges in the scarcity of agricultural image-text paired data through an innovative expert-tuning methodology. Zhou et al. [41] proposed a small-sample learning framework based on pre-trained VLMs to generate refined text descriptions of clustered, selected images of representative disease signs using the Qwen-VL model. This framework aims to enhance classification accuracy for complex crop diseases in small-sample scenarios. However, this approach necessitates validation of cross-domain generalization capabilities and may require substantial computational resources for field deployment. To overcome the limitations of wheat disease diagnosis under natural field conditions, Zhang et al. [42] proposed the Wheat Disease Language Model (WDLM), which combines an enhanced Segment Anything Model (SAM) for feature extraction with Chain-of-Thought (CoT) reasoning to generate interpretable diagnostic logic. The model was optimized using the Wheat Disease Semantic Dataset (WDSD) and exhibited improved performance compared to conventional CNNs, transformers, and existing VLMs in terms of both disease classification accuracy (92.7%) and treatment recommendation relevance. The WDLM achieves practical mobile deployment through computational efficiency optimizations, enabling real-time diagnostics in field applications.

VLMs offer many potential applications in this domain. However, it is also limited by the availability of comprehensive data that reflects the complexities of real-world agriculture. Current research has yet to comprehensively evaluate and study the performance and application of VLMs in agricultural plant classification. To address these challenges and further advance research in precision agriculture, this paper proposes the OpenPlant dataset to offer a valuable resource for precision agriculture researchers. Popular deep learning models and VLMs are evaluated on the dataset to explore the current status and future direction of deep learning and VLMs in plant classification.

## 3. The OpenPlant Dataset

### 3.1. Overview of OpenPlant

Developing an agricultural benchmark dataset should focus on the following factors: taxonomic coverage across species and growth stages, environmental diversity with real-field conditions, community accessibility, and expert-validated annotation to ensure reliability. Based on these rules, we compiled the OpenPlant dataset from a wide range of existing datasets, as shown in Table 1.

#### 3.1.1. Diversity

The OpenPlant dataset exhibits two complementary forms of diversity: inter-class and intra-class diversity. Inter-class diversity means a broad taxonomic range, encompassing 48 orders, 125 families, and 428 genera within the plant kingdom. This taxonomic range is more extensive than many previous agricultural datasets focused on a limited number of crops or weeds. The dataset covers economically important crops such as cereals, legumes, fruits, and vegetables. It also includes weed species, covering both widespread agricultural weeds and emerging invasive taxa. A notable feature of the dataset is the inclusion of uncommon plant taxa in agricultural landscapes, such as wild plants, flowers, ornamental plants, medicinal plants, and multi-purpose plants. This complements most datasets that focus only on specific crops or weeds. It also supplies negative samples for model training to enhance accuracy in complex field environments.

**Figure 2:**
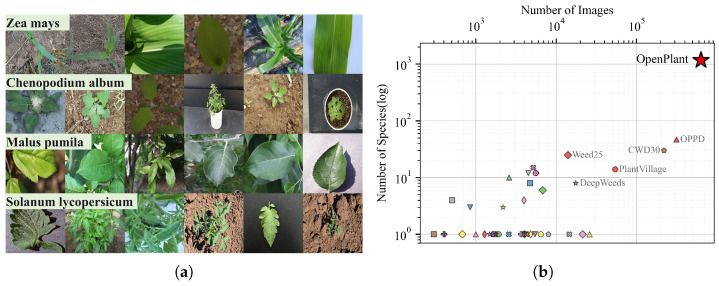
Diversity and richness of the OpenPlant. (**a**) Representative plant species across datasets. Each row corresponds to one species, with images from different datasets. (**b**) Species and image quantity in agricultural datasets. Each marker represents one dataset in Table 1. The x-axis shows the number of images (log scale), and the y-axis shows the number of species (log scale).

Equally important is the intra-class diversity, i.e., visual variation within a single species, including differences in growth stages, shooting angles, environmental conditions (e.g., background, lighting), cultivation settings, and scale (from zoomed-out views of whole plants or leaves to close-up views of local structures). As illustrated in Figure 2a, in OpenPlant dataset, images in one species may source from multiple repositories. This enables the intra-class diversity across growing stages, imaging perspectives, environmental conditions, and cultivation contexts. It is rarely available from a single source collection.

#### 3.1.2. Richness

The OpenPlant dataset represents a significant advancement in the scale of agricultural vision data, encompassing 635,176 images across 1167 plant species. This collection substantially exceeds existing agricultural datasets, as illustrated in Figure 2b. OpenPlant shows a leading position. Compared with most existing agricultural datasets, it offers about 100 times greater species coverage and 2 to 10 times more images. Despite following a long-tail distribution, OpenPlant ensures sufficient data across species. Each species has an average of 544 images, exceeding the minimum thresholds for effective deep learning model training. The head portion of the dataset contains significant crops and common weeds in agriculture, averaging 2430 images per species. For example, the image collection comprises 10,981 images of *Zea mays* (corn) and 7750 images of *Chenopodium album* (fat-hen, goosefoot), capturing diverse growth stages and cultivation environments. The richness of the dataset enables models to develop robust feature identification capabilities that address the significant visual variability in real-world agricultural applications.

#### 3.1.3. Hierarchy

**Figure 3:**
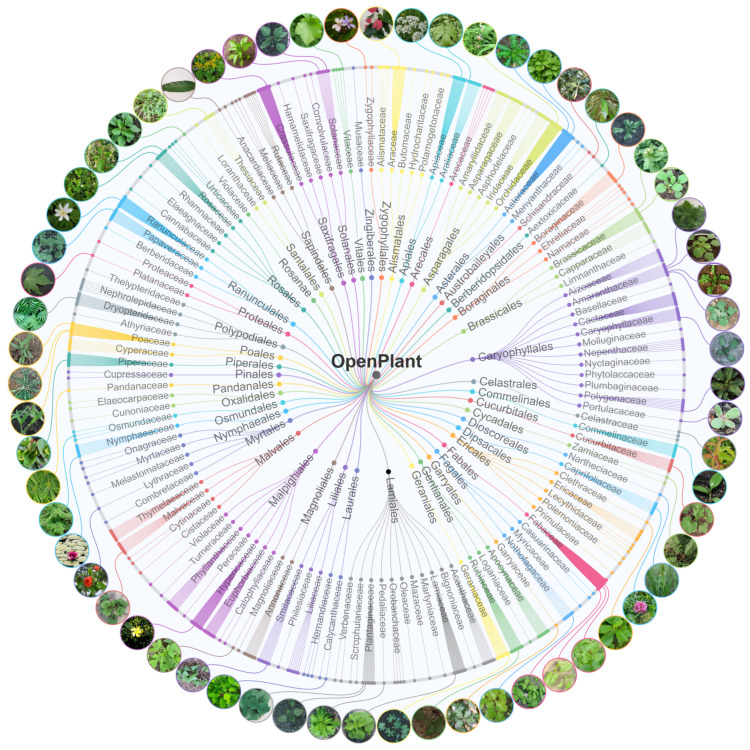
Hierarchical structure of the OpenPlant dataset (order, family, species). The outer circle shows example images. Different colors represent different plant orders. The genera level was hided.

We organized images according to plant taxonomy (order, family, genus, species) to ensure hierarchical classification. This hierarchical system helps in the detailed analysis of specific plant species and broader investigations of taxonomic characteristics. The hierarchical structure of OpenPlant dataset is shown in Figure 3. This circular dendrogram illustrates the taxonomic range of our collection. The rings represent taxonomic levels: orders (innermost ring), families, and species (outermost ring). The visualization shows the broad coverage of OpenPlant, encompassing 48 orders, including major agricultural groups such as Poales, Fabales, Rosales, and Solanales. These orders branch into 125 families, which are further subdivided into 428 genera and 1167 species. Images of representative species along the outermost layer illustrate the important crops and weeds within each taxonomic branch.

This hierarchical organization provides significant advantages for deep learning applications. It helps to understand the relationships of plants at different taxonomic levels (order, family, genus, species). At the same time, it also provides a framework for developing deep learning algorithms based on plant classification hierarchy. This extensive collection provides essential training data for models to recognize plant relationships across different levels of detail.

### 3.2. Data Collection

#### 3.2.1. Dataset Integration

The OpenPlant dataset was created by integrating 41 existing public datasets. From the open-access datasets, we selected 24 disease classification datasets (only healthy samples), 16 datasets focused on weed identification, and one general plant dataset. The collection process used a structured approach to ensure data quality and taxonomic accuracy. This integration required extensive preprocessing to maintain data integrity and avoid duplication.

The data acquisition strategy aimed to enhance botanical and geographical representation by including diverse sources. We checked datasets from agricultural research institutes, universities, and reputable platforms. The selection criteria were: (1) credibility and research impact of the source, (2) dataset size and taxonomic coverage, (3) annotation quality, (4) geographical diversity, and (5) licensing compatibility with open access. Ultimately, the integrated dataset selected 3 datasets from professional agricultural websites, 12 from online platforms, and 29 released with literature. During this process, we did not include several potentially relevant datasets due to specific reasons. For example, we excluded the CropAndWeed dataset due to annotation quality, including repeated labels and taxonomic errors. We did not include datasets such as AIWeeds [67], RumexWeeds [68], and the CNU Weeds datasets [69] because of access restrictions or incomplete data availability. We excluded the Leaf Counting Dataset [31] because it was classified by leaf count instead of taxonomic category. We also did not include datasets like the Maize-Weed Image Dataset [70] and DeepSeedling. These datasets lack species-level labels and group all weeds into a generic “weed” category without any taxonomic differentiation.

The dataset will undergo ongoing maintenance to add new public datasets that meet the criteria. This ensures OpenPlant remains up-to-date with evolving agricultural image data for species classification.

#### 3.2.2. Data Cleaning and Preprocessing

To enhance the usability of OpenPlant, we processed all datasets in Table 1. Most agricultural datasets for disease identification, like PlantVillage and OLID I, contain mixed healthy and diseased leaves, with varying levels of documentation and preprocessing. We selected only healthy samples from these to include in the dataset.

At the dataset level, data overlaps were found in collections such as CWID15 and CWD12, developed by the same research team. These overlapping data were removed during merging to prevent potential data leakage between the training and testing sets. At the file level, some samples in datasets such as PLD and Chilli have identical filenames across different folders but contain distinct image content. After verification of their distinctiveness, these images were retained in our collection.

We applied strict quality criteria to decide which images to include in our final dataset. For time-series datasets recording plant growth from germination (e.g., CWD30), images where the target species is invisible or barely visible during the early growth stages must be carefully screened and removed. For object detection datasets like ImageWeeds and SugarBeet2016, we extracted object regions using the provided bounding box annotations. We excluded bounding boxes smaller than 128 × 128 pixels because they provided insufficient visual information for reliable feature extraction. Quality control also involves identifying and correcting annotation errors through detailed manual review to ensure the datasets maintain a high level of accuracy.

#### 3.2.3. Taxonomic Organization

We performed a standardization process in building the OpenPlant dataset to ensure its structure conforms to the taxonomic system. One major difficulty in our workflow was that many existing agricultural datasets categorize plant species using common names (e.g., “shepherd’s purse”, “barnyard grass”) instead of scientific nomenclature. These common names often vary regionally and may refer to multiple distinct species. During the preprocessing phase, we converted all common names to their corresponding scientific names (genus and species) to ensure taxonomic precision and applicability. This conversion process involved cross-validating multiple botanical databases, such as the World Flora Online (Available online: http://www.worldfloraonline.org, accessed on 1 May 2025) and the Global Biodiversity Information Facility (Available online: https://www.gbif.org, accessed on 1 May 2025).

While working with PlantNet-300K, originally including 1081 species classes, we identified 60 duplicate classes due to different citation styles and incorrect format. For example, “*Cirsium palustre* (L.) Coss. ex Scop.” and “*Cirsium palustre* (L.) Scop.” were incorrectly treated as separate species due to different scientific names with author citation. Similarly, “*Acalypha hispida* Burm. f.” and “*Acalypha hispida* Burm.f.” were mistakenly regarded as different species due to an extra space in the name of the former. We compared the taxonomic information with the WFO Plant List database and integrated it into 1021 categories.

In the OpenPlant dataset, we standardized species names using the binomial nomenclature. Where taxonomic assignment was unclear, we conducted manual checks to enhance accuracy and consistency in our taxonomy. Each image was placed in a folder named according to accepted Latin nomenclature (*Genus species*) for its species. This standardization will simplify the integration with more agricultural plant databases that use scientific nomenclature in the future.

## 4. Methods

### 4.1. Model Selection

**Figure 4:**
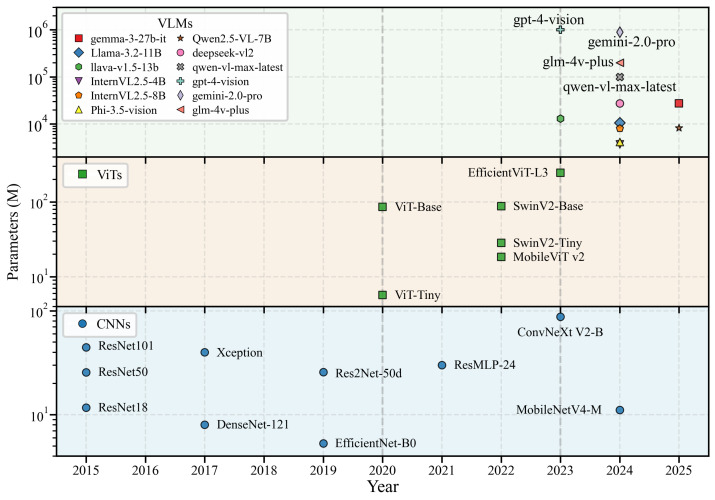
Evolution and Parameter Scale of Models Evaluated in OpenPlant.

We selected models that demonstrated strong capabilities, in general, image classification tasks to evaluate their performance in agricultural plant classification. This provide a useful foundation for evaluating their performance on the OpenPlant dataset and developing detailed performance benchmarks for agricultural plant classification. The selection criteria focused on architectural diversity, historical performance in image classification, evolution of models, and availability of existing deep learning frameworks. Figure 4 shows a timeline of the 28 models benchmarked in our study, grouped into three categories: CNNs, ViTs, and VLMs. This figure includes publication dates and parameter counts, showing the architectural evolution since 2015 and the scale trends on a logarithmic scale.

### 4.2. Computer Vision Models

We tested a broad range of CNN and ViT models using the PyTorch Image Models (timm 1.0.20) library as shown in Table 2.

Convolutional Neural Networks: The model selection was based on their effectiveness in image classification. We began with the ResNet family, which introduced residual learning to train deeper networks effectively. This technique has notably influenced subsequent model designs due to its effectiveness in addressing vanishing gradients. Xception uses depthwise separable convolutions to improve parameter efficiency and processing speed. DenseNet establishes dense connections between all preceding layers and subsequent layers to reduce vanishing gradients and improve model performance. EfficientNet, developed in 2019, uses compound scaling to balance network depth, width, and resolution to enhance performance. Res2Net improves handling multiple scales within a network by using a novel multi-scale feature extraction scheme within residual blocks. ResMLP uses a multi-layer perceptron (MLP)-based feature extraction without conventional convolutions, introducing an alternative approach in network design. MobileNetV4 is designed for environments with limited computational resources, making it ideal for real-time applications. ConvNeXtV2 updates CNNs by integrating transformer-inspired modifications.

Vision Transformers: We chose vision transformer models on various complexity levels, including ViT-Base and ViT-Tiny. These models allow us to investigate how different model sizes affect performance and accuracy. We also included hierarchical designs such as SwinV2-Base and SwinV2-Tiny. These are particularly useful for handling images with varying spatial resolutions, a common scenario in agricultural applications.

The computing power available varies across different application scenarios. The MobileViTV2 has a smaller model size and is well-suited for environments with limited computational resources. EfficientViT-L3, with a larger parameter count, making it appropriate for scenarios where more complex processing is feasible. This contrast allows us to explore the balance between efficiency and performance. It is crucial for practical applications in agricultural settings.

### 4.3. Vision Language Models

To assess the potential capabilities of large multimodal models in agricultural plant classification, we evaluated 12 VLMs, including both open-access and closed-source models, in Table 3.

Open-source VLMs: We selected a diverse set of open-source vision-language models for comprehensive comparative analysis. The Gemma-3 model was quantized using quantization-aware training (QAT) and 4-bit weight quantization. This approach reduced memory usage to about one-third of the original requirement while maintaining comparable accuracy and computational efficiency. Llama-3.2-11B-Vision exhibits solid performance in visual recognition and image reasoning, capable of identifying and analyzing complex patterns. LLaVA-1.5-13B designed a transformer-based architecture with an image encoder and projection layers. These components align visual embeddings with the language model space, enabling efficient multimodal inference. InternVL2.5-4B/8B employed a ViT-MLP-LLM architecture, where ViT extracts visual features, MLP maps them to the language model’s embedding space, and LLM generates the responses. Phi-3.5-Vision is capable of processing high-resolution, information-dense images and corresponding textual inputs, and producing multimodal outputs. Qwen2.5-VL is effective at visual recognition and generating structured outputs from complex visual data.

Closed-source VLMs: To better understand the performance limits, we tested our dataset using several leading closed-source vision-language models. These models are developed commercially and are trained with larger and more diverse resources than most open-source models. In our evaluation, we included Qwen-VL-Max, GPT-4V (GPT-4-vision), Gemini-2.0 (Gemini-2.0-Pro), GLM-4V-Plus, and DeepSeek-VL2. This comparison helps us see how open-source models perform relative to advanced commercial models.

Overall, model selection focuses on architecture innovation, model performance, and practical application in agriculture, ensuring a comprehensive evaluation of diverse paradigms and technologies.

## 5. Experimental Setup

### 5.1. Training of Computer Vision Models

To evaluate plant classification models, all models were initialized with pre-trained weights from ImageNet to make use of the benefits of transfer learning. The final classification layer of each model was replaced with a new fully connected layer corresponding to our dataset’s class count. We resized all images to maintain a fixed shorter dimension of 256 pixels while preserving the original aspect ratio. This approach was intended to preserve the aspect ratio features of the images while standardizing the input dimensions for the neural networks. The dataset was randomly partitioned using a 7:1:2 split ratio for training, validation, and testing sets, respectively. The input images were normalized using a mean of [0.43056735, 0.46167394, 0.36951741] and a standard deviation of [0.22340974, 0.21595199, 0.22381605].

### 5.2. VLM Testing

The evaluation aimed to investigate the performance of VLMs in identifying plant species. It placed special emphasis on the accurate use of scientific names, a task that is uniquely challenging compared to conventional object recognition.

We designed a hierarchical evaluation method to assess the performance of visual language models across varying levels of difficulty. Based on the results of computer vision models, we divided test images into three categories.

- Easy samples: Images that all 16 computer vision models classify correctly.
- Intermediate samples: Images where the classification accuracy of computer vision models is 50%.
- Hard samples: Images that all 16 computer vision models classify incorrectly.

This division enables the evaluation of VLMs’ performance at different identification difficulty levels within traditional vision models.

For open-source VLMs, we obtained the pre-trained models and deployed them locally. For closed-source VLMs, we submitted images via commercial APIs to obtain outputs. We processed the test images from the dataset using the same evaluation pipeline to ensure consistency.

In our tests, when the output format was not fixed, the VLMs often produced answers in various formats. It is difficult for us to accurately count the results. Therefore, we adopted a single-choice evaluation framework. For each test image, we extracted the top-5 predictions with the highest score from the test results of CNN/ViT models based on the highest probability score for the true label. If none of the top-5 predictions contain the true label, the true label was added to five options and replaced the label with the lowest probability. These five candidate labels were then submitted to VLMs as options A to E.

The VLMs were prompted using the same expert-role instruction template. Images are submitted via Base64 encoding. The user prompt instructs the VLMs to analyze the visible features of the image and select the most appropriate option from the five provided categories. This approach limited the VLMs to choosing from predefined options rather than generating free-form taxonomic names. Standardized answers make it possible to assess their visual reasoning abilities. Notably, although the output format was restricted, some models still returned an “ERROR” option for a small number of samples during testing. These cases were counted as incorrect predictions. All VLMs’ responses were parsed to extract their selections and corresponding confidence scores.

### 5.3. Evaluation Metrics

To comprehensively evaluate the model’s performance on the OpenPlant dataset, we employed a diverse suite of evaluation metrics covering classification accuracy, calibration, and inter-model consistency.

For classification assessment, we calculated standard metrics including Precision, Recall, F1-score, and Top-5 accuracy.

We also evaluated the models using Micro-average precision (Micro-AP), Macro-average precision (Macro-AP), and Top-1 Accuracy. Top-1 accuracy was computed separately for the head, medium, tail and the entire dataset. This helps compare the model’s performance on common and rare species.

We calculated the area under the receiver operating characteristic curve (ROC-AUC). The ROC plots the true positive rate (TPR) against the false positive rate (FPR) at different classification thresholds. The AUC reflects the model’s ability to distinguish between classes.

Cohen’s Kappa coefficient quantified the agreement between model predictions and ground truth beyond chance:

(1) $$\begin{array}{c} Kappa=\frac{p_{o}-p_{e}}{1-p_{e}} \end{array}$$

where $p_{o}$ is the observed agreement and $p_{e}$ is the expected agreement by chance.

We calculated the Expected Calibration Error (ECE) for VLMs to measures the gap between a model’s predicted confidence and its actual accuracy:

(2) $$\begin{array}{c} ECE=\sum_{m=1}^{M}\frac{|B_{m}|}{n}\left|\mathrm{acc}\left(B_{m}\right)-\mathrm{conf}\left(B_{m}\right)\right| \end{array}$$

where samples are grouped into *M* bins $B_{m}$ based on prediction confidence, and acc($B_{m}$) and conf($B_{m}$) represent the accuracy and average confidence within each bin.

In addition to individual evaluation metrics above, we calculated an overall performance score using the Entropy Weight Method (EWM).

Let $S_{i,j}$ denote the original score of model *i* on metric *j*. We normalize all metrics to eliminate the influence of different scales:

(3) $$\begin{array}{c} S_{i,j}^{\prime }=\frac{S_{i,j}-\min_{i}\left(S_{i,j}\right)}{\max_{i}\left(S_{i,j}\right)-\min_{i}\left(S_{i,j}\right)} \end{array}$$

We calculate the proportion of model *i* on metric *j*:

(4) $$\begin{array}{c} p_{i,j}=\frac{S_{i,j}^{\prime }}{\sum_{i=1}^{m}S_{i,j}^{\prime }} \end{array}$$

The information entropy $e_{j}$ of the *j*-th metric is defined as follows:

(5) $$\begin{array}{c} e_{j}=-k\sum_{i=1}^{m}p_{i,j}\ln\left(p_{i,j}\right),\;k=\frac{1}{\ln m} \end{array}$$

The entropy weight $w_{j}$ for each metric is then

(6) $$\begin{array}{c} w_{j}=\frac{1-e_{j}}{\sum_{j=1}^{n}\left(1-e_{j}\right)} \end{array}$$

The overall performance score ${\mathrm{Score}}_{i}$ for model *i* is obtained by

(7) $$\begin{array}{c} Score_{i}=\sum_{j=1}^{n}w_{j}\cdot S_{i,j}^{\prime } \end{array}$$

This method tends to assign higher weights to metrics with greater variability across models, thereby allowing such metrics to contribute more to the overall score.

Finally, to evaluate consistency across model architectures, we computed Pearson correlation coefficients between model prediction vectors:

(8) $$\begin{array}{c} r=\frac{\sum_{i=1}^{n}\left(x_{i}-\bar{x}\right)\left(y_{i}-\bar{y}\right)}{\sqrt{\sum_{i=1}^{n}{\left(x_{i}-\bar{x}\right)}^{2}\sum_{i=1}^{n}{\left(y_{i}-\bar{y}\right)}^{2}}} \end{array}$$

where $x_{i}$ and $y_{i}$ are prediction scores from two different models. This metric reveals architectural biases and provides insight into ensemble potential, as models with complementary error patterns offer greater performance gains when combined.

## 6. Results

### 6.1. CNN and ViT Models

We conducted a detailed evaluation of 16 deep learning models on the OpenPlant dataset. The quantitative results indicate substantial performance variations across models and metrics.

**Figure 5:**
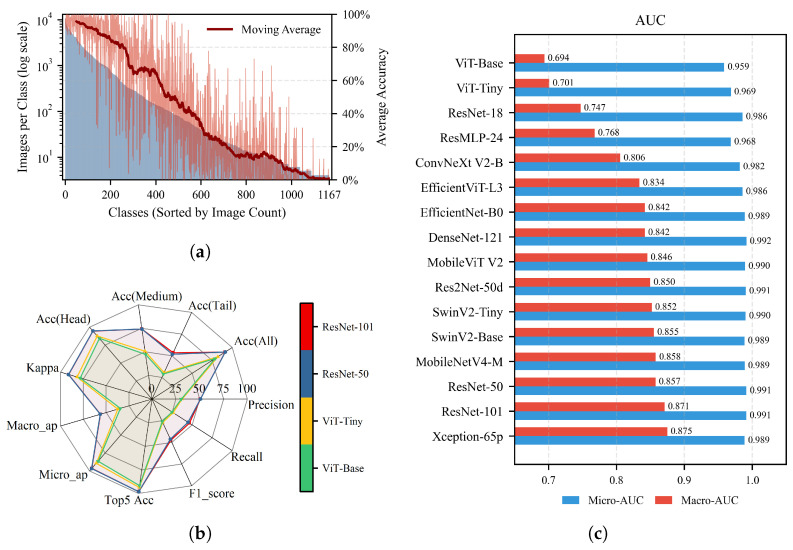
The evaluating results of CNNs and ViTs. (**a**) Training data distribution and its relation to recognition accuracy. The blue area shows the number of images per class (log scale), sorted by image count. The red curve shows the 50-class moving average of recognition accuracy. (**b**) Performance Comparison of CNN and ViT Models. The radar chart shows the results of 16 models across 11 metrics based on OpenPlant dataset and same evaluation settings. (**c**) Micro vs. Macro AUC performance comparison across 16 models.

Figure 5a shows the relationship between the training sample quantity and the recognition accuracy for 1167 plant species. The plot compares the number of images for each class with the accuracy achieved by the models. A discernible trend can be observed: species with more images tend to achieve higher accuracy. There is a positive Pearson correlation (r = 0.5516) between total image count and average accuracy. When the number of images drops below approximately 100, accuracy tends to decrease more noticeably. This pattern suggests that having enough images is important for training deep learning models in plant recognition.

Table 4 and Figure 5b compare 16 CNN and Transformer models on 11 metrics. Overall, CNNs performed better than pure vision transformers. Among all models, ResNet-101 (Score = 96.95%) ranked highest, while DenseNet-121 showed the best accuracy (91.37%). Pure ViT models, such as ViT-Base, had lower F1-scores (25.91%) than CNNs (e.g., Xception-65p: 48.31%) and other ViTs(e.g., SwinV2-Base: 46.43%), indicating weaker performance on long-tailed data.

Performance varied greatly between head, medium, and tail classes. The accuracy for head classes was 84–95%, but for tail classes, it dropped to 29–53%. ResNet-101 and Xception achieved the higher accuracy on tail classes (53.39% and 53.18%), indicating their stronger ability in recognizing rare species.

Overall accuracy was high (88–91%), but the gap between micro-AP and macro-AP (about 40%) shows the strong class imbalance effect. Top-5 accuracy was above 96% for most models, meaning the correct label was usually in the top-5 predictions. Kappa values ranged from 78% to 91% and DenseNet-121 achieved highest score.

Figure 5c contrasts micro-AUC and macro-AUC scores for all 16 evaluated models. The notable performance difference reflects the challenge posed by the long-tailed species distribution. All models achieve relatively high micro-AUC values (0.959–0.992), indicating strong performance on common species. However, their lower macro-AUC scores (0.694–0.875) suggest challenges in classifying rare species. Xception-65p and ResNet-101 achieve the highest macro-AUC values (0.875 and 0.871), indicating that these models may capture features more effectively under class imbalance conditions.

**Figure 6:**
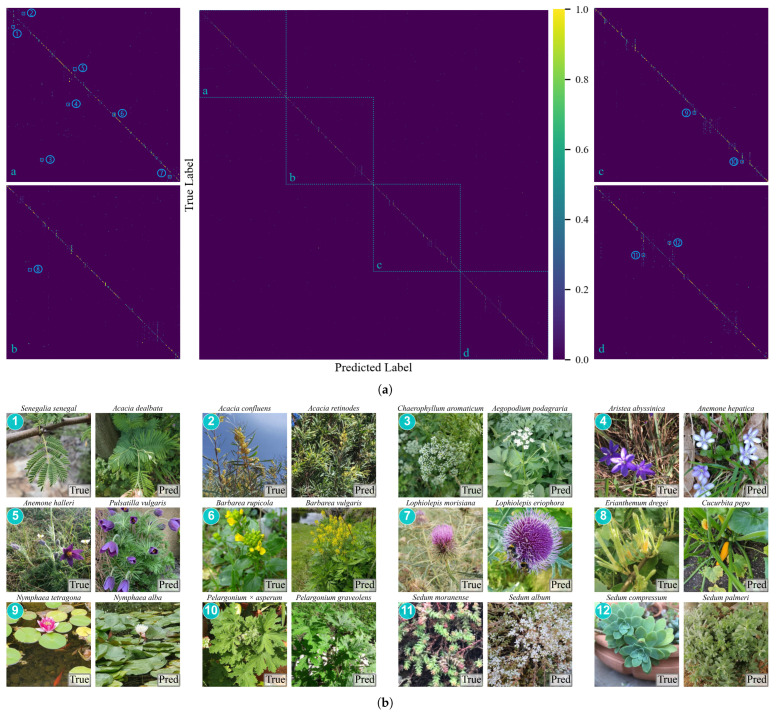
Confusion matrix and examples of misclassified classes. (**a**) Confusion matrix based on test results from 16 models. The main diagonal is divided into four sections (a–d), and each section is zoomed in. Twelve confused class pairs are marked with numbers. (**b**) Example images for each confused pair from (**a**), showing the true label (True) and the predicted label (Pred).

Figure 6 shows the classification results for all 1167 species using the 16 CNN and ViT models. For clearer presentation, the confusion matrix is divided into four enlarged sections (a–d) in Figure 6a. The horizontal axis represents the predicted label, and the vertical axis represents the true label. Brighter yellow indicates higher recognition performance. Examples 1–12 in Figure 6b illustrate the confusion cases labeled in Figure 6a. Many occur between species with very similar leaves or flowers, such as ferns with similar leaves, and thistles with similar flowers. This indicates that the model has learned useful phenotypic features but still performs poorly when handling very subtle differences, particularly among rare or visually similar species. Without careful observation of the details, these similarities may also pose challenges to experienced botanists.

### 6.2. Vision-Language Models

In addition to traditional deep learning architectures, we also evaluated 12 popular VLMs on the OpenPlant dataset. These multimodal models can offer complementary advantages to pure vision models in this challenging task.

**Figure 7:**
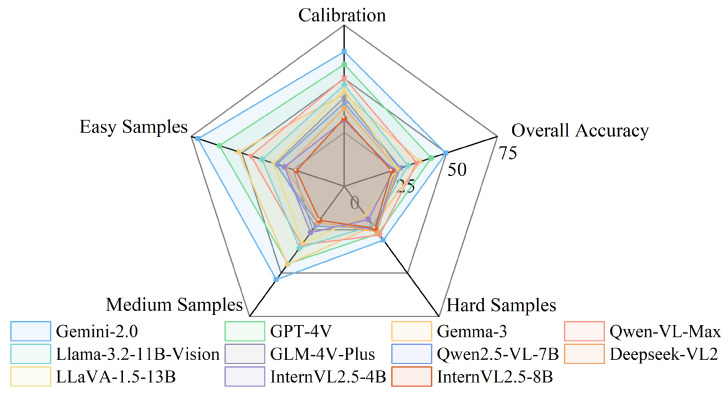
Radar chart of vision-language model performance. Results are based on Table 5. The chart compares Overall Accuracy, performance on Easy, Medium, and Hard samples, and Calibration scores. Calibration is calculated as 1-ECE, so higher values indicate better calibration.

To evaluate the performance of visual language models on samples of varying difficulty, we divided the dataset into three subsets: Hard Samples, Intermediate Samples, and Easy Samples. The evaluation results are shown in Table 5 and Figure 7 illustrating notable performance variations across VLMs.

Gemini-2.0 achieved the highest overall accuracy of 49.70%, followed by GPT-4V (42.60%) and Gemma-3 (37.20%), consistent with their ranking in overall performance score. This performance variation remained relatively consistent across different evaluation metrics. It indicates inherent differences in the visual recognition capabilities of these models rather than task-specific variations.

The expected calibration error (ECE) metrics reveal that most VLMs exhibit poor calibration, with values ranging from 37% (Gemini-2.0) to 69% (InternVL2.5-4B). Lower ECE values indicate better alignment between model confidence and actual accuracy. Gemini-2.0 and GPT-4V showed better calibration than other models. This suggests their confidence scores are more reliable indicators of prediction accuracy.

Across all VLMs, we observed the clear correlation between sample difficulty and model performance. Accuracy on Easy Samples (23–71%) was consistently higher than on Hard Samples (16–30%). This indicates that VLMs struggle with the same challenging cases as pure vision models. However, for 2554 hard samples(incorrectly predicted by 16 CNNs and ViTs), VLMs achieved more than 50% accuracy in 384 samples. This complementary advantage suggests that combining VLM with traditional deep learning models may yield potential benefits.

**Figure 8:**
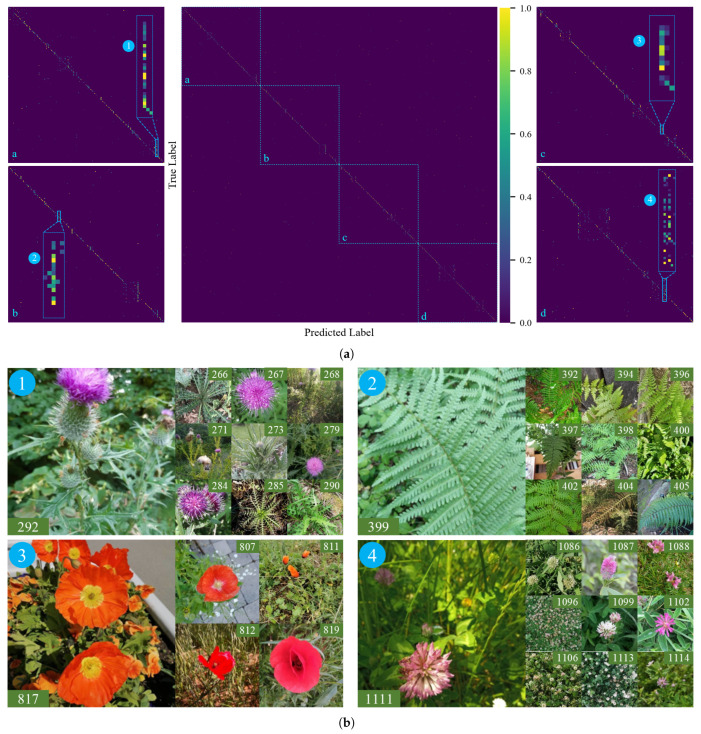
Confusion matrix and example samples from confused regions in VLMs. (**a**) Confusion matrix from VLM results. The main diagonal is divided into four parts (a-d), each part is zoomed in. Four confused regions are marked with numbers. (**b**) Example samples from each confused region in (**a**), with true label shown on the images.

The confusion matrix in Figure 8a shows that the VLMs perform well on most classes. Many errors occur between classes with similar visual features, such as thistle with spiny leaves and purple flowers, where the model focuses more on these dominant features. As shown in Figure 8b, class 292 corresponds to *Cirsium vulgare*, which is a common thistle species. Other species that the model predicts as *Cirsium vulgare* belong to the same genus. If their traits are not sufficiently represented during training, the model is more likely to classify them as the species with a higher sample frequency. In addition, most confused samples in Figure 8b occur within the same genus. Misclassification frequently occurs within a genus because intra-class similarity makes it hard to extract stable features. Future work could use a hierarchical classification approach. The model predicts the correct genus first and then uses more morphological or phenotypic features to identify the correct species.

## 7. Discussion

The different performance in our experiments indicates that the long-tailed distribution with 1167 classes brings challenges for these deep learning models. Models tend to learn features of common species, but their performance declines when classifying rare species with limited training samples. The visual similarity between related species increases the complexity of this task. Phenotypic differences are relatively minor and are often obscured by variations in imaging conditions and growth stages. Figure 6 further illustrates this point. Many errors occur between species with very similar leaves or flowers. This indicates that current models still struggle to capture the fine-grained features needed for reliable recognition of rare or visually similar species.

Future research could focus on developing specialized techniques to improve rare species or similar recognition.Potential approaches include data augmentation for tail classes, transfer learning, hierarchical classification strategies, or combination of different architectures. Another approach is to develop more fine-grained visual feature extraction techniques to capture subtle variations between plant categories. Additionally, integrating multi-source data from sensing platforms, such as RGB imaging or hyperspectral cameras, may help improve classification accuracy.

For vision-language models (VLMs), a similar set of challenges emerges. Since we cannot obtain the distribution information of the pre-training data for VLMs, we cannot directly establish a correlation between accuracy and the sample frequency in the test set. Analysis of performance across the 1167 plant classes revealed that VLMs’ accuracy showed weak correlation with the sample frequency of the OpenPlant dataset. For example, VLMs achieved 100% accuracy on certain categories containing only 4–9 images. At the same time, there are also categories with a large number of test samples where VLM’s accuracy remains relatively low. In fact, VLMs are also influenced by similar phenotypic characteristics among different species within the same taxonomic group.

**Figure 9:**
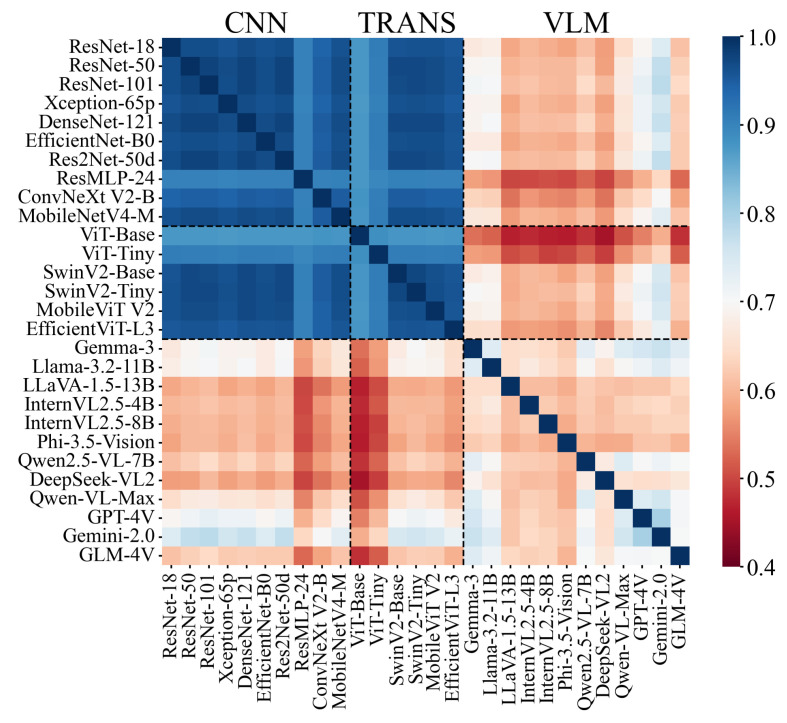
Inter-model correlation heatmap showing test result similarity among CNNs, Transformers, and vision-language models (VLMs) for agricultural species classification based on OpenPlant.

To assess the consistency across all models, we computed Pearson correlation coefficients based on prediction results. Figure 9 illustrates prediction correlations among 28 models including CNNs, ViTs, and VLMs evaluated on the OpenPlant dataset. Higher correlation indicates that two models tend to produce more similar prediction results. Different correlations emerge between model families. CNNs and ViTs exhibit high correlations within groups, indicating that these models capture similar features. Therefore, CNNs and ViTs can serve as alternative solutions to each other, and the performance gains from integrating the two may be limited. In contrast, VLMs exhibit lower correlations (0.4–0.7) compared to CNNs and ViTs. Combining VLM with CNNs and ViTs may enhance model robustness and species identification through capturing complementary features in complex agricultural scenarios. Additionally, agricultural VLMs could be developed to enhance species recognition accuracy and support data-driven decision-making in farming.

These observations highlight promising directions for future work. First, combining VLMs with CNNs and ViTs may enhance overall model robustness and species identification performance by leveraging complementary strengths across architectures. Second, specialized VLMs could be developed to reduce misclassification and support more reliable, fine-grained species recognition. Finally, building large datasets can help develop specialized large models to support data-driven decision-making in the agricultural field.

## 8. Conclusions

In this study, we introduced the OpenPlant dataset, a large and diverse agricultural benchmark with 635,176 images across 1167 plant species. It covers a wide range of crops, weeds, and wild plants, including rich variations in growth stages, backgrounds, and environmental conditions. We tested 16 CNN/ViT models and 12 VLMs to evaluate their performance characteristics on the long-tailed dataset.

From our experiments, several key findings emerged: (1) the OpenPlant dataset provides diverse and rich samples of species with hierarchical organization, making it a potentially valuable baseline for agricultural vision tasks, (2) CNN models show stronger and more stable performance than pure ViTs on this long-tailed distributions, with ResNet-101 achieving the highest overall performance scores, (3) the tiny versions of ViT and SwinV2 performed better than their base versions. This may be because large models tend to classify low-frequency samples into high-frequency categories, as indicated by the higher accuracy of tiny versions on tail classes, (4) closed-source VLMs such as Gemini-2.0 and GPT-4V perform better than other VLMs tested in this work, (5) the low correlation between errors of CNN/ViT models and VLMs indicates that these model families focus on different visual features, and (6) the complementarity between model families highlights the potential for integrating VLMs with CNNs and ViTs.

At the same time, the study has some limitations and potential directions: (1) the dataset exhibits long-tail imbalance, which may limit recognition of rare species, and (2) integrating different models, particularly those designed for long-tail data, requires more exploration. Future research could focus on reducing long-tail bias through data augmentation, combining CNNs and ViTs with VLMs, and building specialized agricultural foundation models to enhance the efficiency, effectiveness, and generalization of agricultural AI systems.

## Figures and Tables

**Table 1:** Comparison of public agricultural plant datasets included in the OpenPlant dataset.

Dataset	Classes	Species	Images	Scale	Environment	Target Species	Citation
AppleLeaf9	9	1	14,582	Single-branch	Outdoor	Apple	[11]
ATLDSD	5	1	1641	Single-branch	Outdoor	Apple	[43]
Banana Leaf Disease Images	3	1	1288	Single-leaf	Outdoor	Banana	[24]
Carrot-Weed	2	1	401	Whole-plant	Outdoor	Carrot, weed	[30]
Cassava Disease Classification	5	1	21,400	Single-branch	Outdoor	Cassava	[21]
CGIAR CVCD	3	1	1486	Mixed	Outdoor	Wheat	[44]
Chilli Dataset	5	1	1932	Mixed	Outdoor	Chilli	[45]
Coffee Plant Disease	3	1	1000	Mixed	Outdoor	Coffee	[46]
Corn Leaf Disease	4	1	4000	Single-leaf	Controlled	Corn	[47]
Cotton Plant Disease	6	1	26,100	Mixed	Outdoor	Cotton	[22]
Cotton Leaf Disease Dataset	4	1	1711	Mixed	Outdoor	Cotton	[20]
CottonWeedDet12	12	12	5648	Whole-plant	Outdoor	12 Weeds	[29]
CottonWeedDet3	3	3	848	Whole-plant	Outdoor	3 Weeds	[48]
CottonWeedID15	15	15	5187	Whole-plant	Outdoor	15 Weeds	[27]
Cucumber Plant Diseases	2	1	679	Mixed	Outdoor	Cucumber	[49]
CWD30	30	30	219,770	Whole-plant	Mixed	10 Crops, 20 weeds	[33]
DeepWeeds	8	8	17,509	Whole-plant	Outdoor	8 Weeds	[26]
Early crop–weed	4	4	508	Whole-plant	Outdoor	2 Crops, 2 weeds	[50]
ESCA-dataset	2	1	1768	Single-branch	Outdoor	Grape	[51]
Fruit Leaf	12	12	4503	Single-leaf	Controlled	12 Crops	[52]
ImageWeeds	5	4	3975	Whole-plant	Controlled	4 Weeds	[53]
MangoLeafBD	8	1	4000	Single-leaf	Controlled	Mango	[54]
OLID I	58	8	4749	Single-leaf	Controlled	8 Crops	[55]
OPPD	47	47	315,038	Whole-plant	Controlled	46 Weeds	[13]
Plant Pathology 2020-FGVC7	4	1	3642	Mixed	Outdoor	Apple	[56]
Plant Seedlings	12	12	5545	Whole-plant	Controlled	3 Crops, 9 weeds	[57]
PlantDoc	28	10	2598	Mixed	Mixed	10 Crops	[58]
PlantNet-300K	1081	1081	306,146	Whole-plant	Outdoor	Mixed	[34]
PlantVillage	39	14	54,309	Single-leaf	Controlled	14 crops	[14]
Potato Disease Leaf Dataset	3	1	4062	Single-leaf	Controlled	Potato	[15]
Rice Diseases Image Dataset	4	1	5447	Single-leaf	Controlled	Rice	[23]
SorghumWeedDataset	3	1	4312	Whole-plant	Outdoor	Sorghum, weeds	[59]
SugarBeet2016	2	1	4817	Whole-plant	Outdoor	Sugar beet, weeds	[60]
Sugarcane Disease Dataset	3	1	300	Mixed	Outdoor	Sugarcane	[12]
Sugarcane Leaf Disease	5	1	2569	Single-leaf	Outdoor	Sugarcane	[61]
Soybean Images Dataset	3	1	6410	Whole-plant	Outdoor	Soybean	[62]
TobSet	2	1	8000	Whole-plant	Outdoor	Tobacco, weeds	[63]
VCD	3	3	2182	Whole-plant	Outdoor	Maize, bean, leek	[64]
Weed-datasets	7	6	6793	Whole-plant	Outdoor	Corn, lettuce, weeds	[65]
Weed25	25	25	14,035	Whole-plant	Outdoor	25 Weeds	[28]
Wheat Leaf Dataset	3	1	407	Mixed	Outdoor	Wheat	[66]
OpenPlant	1167	1167	635,176	Mixed	Mixed	Mixed	-

**Table 2:** Comparison of evaluated CNN and ViT models. The table lists the number of parameters (M), FLOPs (G), and a short description of each model’s key architecture features. Models are grouped into CNNs and ViTs.

Model	Params (M)	FLOPs (G)
Convolutional Neural Networks (CNNs)		
ResNet-18	11.7	1.8
ResNet-50	25.6	4.1
ResNet-101	44.5	7.8
Xception-65p	39.8	5.7
DenseNet-121	8.0	2.9
EfficientNet-B0	5.3	0.4
Res2Net-50d	25.7	4.2
ResMLP-24	30.0	6.0
ConvNeXt V2 B	87.7	15.4
MobileNetV4-M	11.1	0.7
Vision Transformers(ViTs)		
ViT-Base	85.8	17.6
ViT-Tiny	5.7	1.3
SwinV2-Base	87.9	15.4
SwinV2-Tiny	28.3	4.5
MobileViTV2	18.4	1.8
EfficientViT-L3	246.0	53.2

**Table 3:** Comparison of evaluated VLMs. The table lists the developer, parameter count, and main features of each model. Open-source and closed-source models are shown separately. Parameter counts for closed-source models are approximate. All listed models can understand images and generate text responses. Specifications are based on public information as of October 2025.

Model	Developer	Specifications
Open-source Models		
Gemma-3	Google	27.4 B params, instruction-tuned, context 8 K, supports multi-language and low-latency inference
Llama-3.2-11B-Vision	Meta AI	10.7 B params, SigLIP vision encoder, context 8 K, strong in visual recognition and reasoning
LLaVA-1.5-13B	Liu et al.	13 B params, CLIP ViT-L/14, context 4 K, aligns image and text for quick multimodal Q&A
InternVL2.5-4B	OpenGVLab	3.71 B params, high-res and video frame input, context 32 K, ViT-MLP-LLM pipeline
InternVL2.5-8B	OpenGVLab	8.08 B params, high-res and multi-image input, context 32 K, handles mixed visual data
Phi-3.5-Vision	Microsoft	4.15 B params, SigLIP encoder, context 8 K, processes dense high-res images well
Qwen2.5-VL-7B	Alibaba Cloud	8.29 B params, high-res support, context 32 K, outputs structured data from visuals
Closed-source Models		
Qwen-VL-Max	Alibaba Cloud	Estimated >100 B params, multi-image reasoning, long context, detailed analysis
GPT-4V	OpenAI	Estimated hundreds of B, advanced visual reasoning, works well on complex multimodal tasks
Gemini-2.0	Google	Estimated >100 B params, multimodal, integrates vision and text with other modalities
GLM-4V-Plus	Zhipu AI	Estimated >100 B params, Chinese-English bilingual, supports diverse multimodal tasks
DeepSeek-VL2	DeepSeek AI	27.5 B params, enhanced visual reasoning, context 8 K, good for document and chart Q&A

**Table 4:** Performance comparison of different CNN and ViT models.

Model	Precision (%)	Recall (%)	F1-Score (%)	Top-5	Micro-AP (%)	Macro-AP (%)	Kappa	Top-1 Accuracy (%)				Score (%)
				**Accuracy (%)**				**Head**	**Medium**	**Tail**	**All**	
Convolutional Neural Networks (CNNs)												
ResNet-18	36.59	33.55	33.87	97.17	95.21	44.51	89.45	94.06	66.23	38.24	89.51	59.09
ResNet-50	50.88	45.06	46.44	98.29	96.61	56.59	91.29	94.74	74.24	51.67	91.34	95.80
ResNet-101	50.82	46.64	47.52	98.28	96.35	56.33	91.11	94.47	**74.29**	**53.39**	91.16	**96.95**
Xception-65p	51.90	**47.43**	**48.31**	97.78	95.50	55.73	89.67	93.14	71.84	53.18	89.73	93.01
DenseNet-121	49.42	43.45	44.73	**98.32**	**96.66**	55.88	**91.32**	**94.87**	73.84	50.26	**91.37**	92.74
EfficientNet-B0	49.51	42.38	44.08	97.83	95.80	54.70	89.93	93.81	70.09	48.54	89.99	85.77
Res2Net-50d	49.63	44.81	45.94	98.12	96.22	54.19	91.08	94.51	74.13	51.76	91.14	92.89
ResMLP-24	40.16	33.43	35.17	93.73	89.29	40.25	81.75	86.51	55.84	38.87	81.86	32.50
ConvNeXtV2 B	43.73	37.06	38.70	96.39	94.03	48.45	87.41	91.82	64.25	43.03	87.49	63.41
MobileNetV4-M	52.06	44.67	46.71	97.82	95.77	55.53	90.07	93.80	70.72	51.22	90.13	91.11
Vision Transformers(ViTs)												
ViT-Base	30.78	24.96	25.91	91.79	86.37	34.46	78.54	84.22	47.26	28.94	78.67	0.00
ViT-Tiny	31.52	26.34	27.04	93.77	89.32	37.49	81.95	87.67	50.66	30.50	82.06	16.04
SwinV2-Base	51.37	44.73	46.43	97.75	95.93	55.52	90.33	94.00	71.70	50.44	90.39	91.18
SwinV2-Tiny	**52.34**	44.44	46.32	98.06	96.34	**58.01**	90.64	94.27	72.26	50.98	90.70	94.21
MobileViTV2	50.86	43.41	45.18	97.93	95.97	55.19	90.29	94.02	71.53	49.11	90.35	89.21
EfficientViT-L3	48.86	41.38	43.33	97.15	95.08	51.79	88.89	92.97	67.47	47.55	88.96	78.86

**Table 5:** Performance comparison of different vision-language models.

Model	Open/Closed	Accuracy (%)							ECE (%)	Score (%)
		**Hard**	**Intermediate**	**Easy**	**Head**	**Medium**	**Tail**	**All**		
Gemini-2.0	closed	**30.97**	**53.77**	**71.60**	**54.98**	**50.72**	**36.47**	**49.70**	**37.37**	**100.00**
GPT-4V	closed	27.21	44.78	61.11	48.02	43.60	29.13	42.60	43.22	74.57
Gemma-3	open	23.00	45.08	51.76	43.67	37.19	23.55	37.20	57.06	53.48
Qwen-VL-Max	closed	28.03	33.72	45.74	39.59	32.41	33.30	35.51	49.48	52.78
Llama-3.2-11B-Vision	open	22.55	35.46	40.23	38.09	29.31	20.40	31.17	52.81	35.80
GLM-4V-Plus	closed	24.67	21.11	33.81	28.39	28.57	23.43	27.50	58.62	22.84
Qwen2.5-VL-7B	open	24.24	22.94	33.00	28.86	26.95	24.67	27.29	61.26	22.04
DeepSeek-VL2	closed	26.04	20.86	28.55	26.07	25.75	27.13	26.15	63.66	19.01
LLaVA-1.5-13B	open	16.57	33.06	34.60	29.72	26.47	16.62	25.89	54.82	19.47
InternVL2.5-4B	open	19.12	26.68	29.19	28.65	22.47	17.68	24.07	69.15	8.63
InternVL2.5-8B	open	24.24	19.58	23.40	23.50	23.07	22.77	23.19	68.27	7.83
Phi-3.5-Vision	open	18.29	29.32	26.77	26.38	23.21	16.41	23.19	66.32	7.83

## Data Availability

The code and data are available on GitHub: https://github.com/Kaiqi6/OpenPlant (accessed on 31 December 2025).

## References

[1] Upadhyay A., Chandel N.S., Singh K.P., Chakraborty S.K., Nandede B.M., Kumar M., Subeesh A., Upendar K., Salem A., Elbeltagi A. (2025). Deep Learning and Computer Vision in Plant Disease Detection: A Comprehensive Review of Techniques, Models, and Trends in Precision Agriculture. Artif. Intell. Rev..

[2] Khan A.T., Jensen S.M., Khan A.R. (2025). Advancing Precision Agriculture: A Comparative Analysis of YOLOv8 for Multi-Class Weed Detection in Cotton Cultivation. Artif. Intell. Agric..

[3] Bosilj P., Duckett T., Cielniak G. (2018). Analysis of Morphology-Based Features for Classification of Crop and Weeds in Precision Agriculture. IEEE Robot. Autom. Lett..

[4] Wu H., Fang L., Yu Q., Yuan J., Yang C. (2023). Plant Leaf Identification Based on Shape and Convolutional Features. Expert Syst. Appl..

[5] Adhinata F.D., Wahyono, Sumiharto R. (2024). A Comprehensive Survey on Weed and Crop Classification Using Machine Learning and Deep Learning. Artif. Intell. Agric..

[6] Li W., Liang S., Zhang Y., Liu L., Chen K., Chen Y., Ma H., Xu J., Ma Y., Guan S. (2025). Fine-Grained Hierarchical Crop Type Classification from Integrated Hyperspectral EnMAP Data and Multispectral Sentinel-2 Time Series: A Large-Scale Dataset and Dual-Stream Transformer Method. arXiv.

[7] Zheng Y.Y., Kong J.L., Jin X.B., Wang X.Y., Su T.L., Zuo M. (2019). CropDeep: The Crop Vision Dataset for Deep-Learning-Based Classification and Detection in Precision Agriculture. Sensors.

[8] Coleman G.R., Bender A., Hu K., Sharpe S.M., Schumann A.W., Wang Z., Bagavathiannan M.V., Boyd N.S., Walsh M.J. (2022). Weed Detection to Weed Recognition: Reviewing 50 Years of Research to Identify Constraints and Opportunities for Large-Scale Cropping Systems. Weed Technol..

[9] Mylonas N., Malounas I., Mouseti S., Vali E., Espejo-Garcia B., Fountas S. (2022). Eden Library: A Long-Term Database for Storing Agricultural Multi-Sensor Datasets from UAV and Proximal Platforms. Smart Agric. Technol..

[10] Weyler J., Magistri F., Marks E., Chong Y.L., Sodano M., Roggiolani G., Chebrolu N., Stachniss C., Behley J. (2024). PhenoBench—A Large Dataset and Benchmarks for Semantic Image Interpretation in the Agricultural Domain. IEEE Trans. Pattern Anal. Mach. Intell..

[11] Yang Q., Duan S., Wang L. (2022). Efficient Identification of Apple Leaf Diseases in the Wild Using Convolutional Neural Networks. Agronomy.

[12] Soundar P. (2022). Sugarcane Disease Dataset. https://www.kaggle.com/datasets/prabhakaransoundar/sugarcane-disease-dataset.

[13] Leminen Madsen S., Mathiassen S.K., Dyrmann M., Laursen M.S., Paz L.C., Jørgensen R.N. (2020). Open Plant Phenotype Database of Common Weeds in Denmark. Remote Sens..

[14] Hughes D.P., Salathe M. (2016). An Open Access Repository of Images on Plant Health to Enable the Development of Mobile Disease Diagnostics. arXiv.

[15] Rashid J., Khan I., Ali G., Almotiri S.H., AlGhamdi M.A., Masood K. (2021). Multi-Level Deep Learning Model for Potato Leaf Disease Recognition. Electronics.

[16] Dong X., Wang Q., Huang Q., Ge Q., Zhao K., Wu X., Wu X., Lei L., Hao G. (2023). PDDD-PreTrain: A Series of Commonly Used Pre-Trained Models Support Image-Based Plant Disease Diagnosis. Plant Phenomics.

[17] Liu X., Min W., Mei S., Wang L., Jiang S. (2021). Plant Disease Recognition: A Large-Scale Benchmark Dataset and a Visual Region and Loss Reweighting Approach. IEEE Trans. Image Process..

[18] Lu Y., Young S. (2020). A Survey of Public Datasets for Computer Vision Tasks in Precision Agriculture. Comput. Electron. Agric..

[19] Li J., Xu M., Xiang L., Chen D., Zhuang W., Yin X., Li Z. (2024). Foundation Models in Smart Agriculture: Basics, Opportunities, and Challenges. Comput. Electron. Agric..

[20] Noon S.K., Amjad M., Ali Qureshi M., Mannan A. (2021). Computationally Light Deep Learning Framework to Recognize Cotton Leaf Diseases. J. Intell. Fuzzy Syst..

[21] Mwebaze E., Gebru T., Frome A., Nsumba S., Tusubira J. (2019). iCassava 2019 Fine-Grained Visual Categorization Challenge. arXiv.

[22] Dhamodharan R. (2023). Cotton Plant Disease. https://www.kaggle.com/datasets/dhamur/cotton-plant-disease.

[23] Do H.M. (2019). Rice Diseases Image Dataset. https://www.kaggle.com/datasets/minhhuy2810/rice-diseases-image-dataset.

[24] Hailu Y. (2021). Banana Leaf Disease Images. https://data.mendeley.com/datasets/rjykr62kdh/1.

[25] Dong X., Zhao K., Wang Q., Wu X., Huang Y., Wu X., Zhang T., Dong Y., Gao Y., Chen P. (2024). PlantPAD: A Platform for Large-Scale Image Phenomics Analysis of Disease in Plant Science. Nucleic Acids Res..

[26] Olsen A., Konovalov D.A., Philippa B., Ridd P., Wood J.C., Johns J., Banks W., Girgenti B., Kenny O., Whinney J. (2019). DeepWeeds: A Multiclass Weed Species Image Dataset for Deep Learning. Sci. Rep..

[27] Chen D., Lu Y., Li Z., Young S. (2022). Performance Evaluation of Deep Transfer Learning on Multi-Class Identification of Common Weed Species in Cotton Production Systems. Comput. Electron. Agric..

[28] Wang P., Tang Y., Luo F., Wang L., Li C., Niu Q., Li H. (2022). Weed25: A Deep Learning Dataset for Weed Identification. Front. Plant Sci..

[29] Dang F., Chen D., Lu Y., Li Z. (2023). YOLOWeeds: A Novel Benchmark of YOLO Object Detectors for Multi-Class Weed Detection in Cotton Production Systems. Comput. Electron. Agric..

[30] Lameski P., Zdravevski E., Trajkovik V., Kulakov A., Trajanov D., Bakeva V. (2017). Weed Detection Dataset with RGB Images Taken under Variable Light Conditions. Proceedings of the CT Innovations 2017.

[31] Teimouri N., Dyrmann M., Nielsen P.R., Mathiassen S.K., Somerville G.J., Jørgensen R.N. (2018). Weed Growth Stage Estimator Using Deep Convolutional Neural Networks. Sensors.

[32] Jiang Y., Li C., Paterson A.H., Robertson J.S. (2019). DeepSeedling: Deep Convolutional Network and Kalman Filter for Plant Seedling Detection and Counting in the Field. Plant Methods.

[33] Ilyas T., Arsa D.M.S., Ahmad K., Lee J., Won O., Lee H., Kim H., Park D.S. (2025). CWD30: A New Benchmark Dataset for Crop Weed Recognition in Precision Agriculture. Comput. Electron. Agric..

[34] Garcin C., Joly A., Bonnet P., Affouard A., Lombardo J.-C., Chouet M., Servajean M., Lorieul T., Salmon J. (2021). Pl@ntNet-300K Image Dataset.

[35] Shah S.R., Qadri S., Bibi H., Shah S.M.W., Sharif M.I., Marinello F. (2023). Comparing Inception V3, VGG 16, VGG 19, CNN, and ResNet 50: A Case Study on Early Detection of a Rice Disease. Agronomy.

[36] Liu W., Yu L., Luo J. (2022). A Hybrid Attention-Enhanced DenseNet Neural Network Model Based on Improved U-net for Rice Leaf Disease Identification. Front. Plant Sci..

[37] Shaheed K., Qureshi I., Abbas F., Jabbar S., Abbas Q., Ahmad H., Sajid M.Z. (2023). EfficientRMT-net—An Efficient ResNet-50 and Vision Transformers Approach for Classifying Potato Plant Leaf Diseases. Sensors.

[38] Sun C., Zhang M., Zhou M., Zhou X. (2024). An Improved Transformer Network with Multi-Scale Convolution for Weed Identification in Sugarcane Field. IEEE Access.

[39] Thakur P.S., Chaturvedi S., Khanna P., Sheorey T., Ojha A. (2023). Vision Transformer Meets Convolutional Neural Network for Plant Disease Classification. Ecol. Inf..

[40] Awais M., Alharthi A.H.S.A., Kumar A., Cholakkal H., Anwer R.M. (2025). AgroGPT: Efficient Agricultural Vision-Language Model with Expert Tuning. arXiv.

[41] Zhou Y., Yan H., Ding K., Cai T., Zhang Y. (2024). Few-Shot Image Classification of Crop Diseases Based on Vision–Language Models. Sensors.

[42] Zhang K., Ma L., Cui B., Li X., Zhang B., Xie N. (2024). Visual Large Language Model for Wheat Disease Diagnosis in the Wild. Comput. Electron. Agric..

[43] Feng J., Chao X. (2022). Apple Tree Leaf Disease Segmentation Dataset.

[44] Hussain S. (2020). CGIAR Computer Vision for Crop Disease. https://www.kaggle.com/datasets/shadabhussain/cgiar-computer-vision-for-crop-disease.

[45] Naik B.N., Malmathanraj R., Palanisamy P. (2022). Detection and Classification of Chilli Leaf Disease Using a Squeeze-and-Excitation-Based CNN Model. Ecol. Inf..

[46] (2021). Coffee Disease. Coffee Plant Disease. https://www.kaggle.com/datasets/coffeedisease/coffee-plant-disease.

[47] Saputra S.I. (2023). Corn Leaf Disease. https://www.kaggle.com/datasets/ndisan/corn-leaf-disease.

[48] Rahman A., Lu Y., Wang H. (2023). Performance Evaluation of Deep Learning Object Detectors for Weed Detection for Cotton. Smart Agric. Technol..

[49] Negm K. (2020). Cucumber Plant Diseases Dataset. https://www.kaggle.com/datasets/kareem3egm/cucumber-plant-diseases-dataset.

[50] AUAgroup (2024). AUAgroup/Early-Crop-Weed. https://github.com/AUAgroup/early-crop-weed.

[51] Alessandrini M., Calero Fuentes Rivera R., Falaschetti L., Pau D., Tomaselli V., Turchetti C. (2021). A Grapevine Leaves Dataset for Early Detection and Classification of Esca Disease in Vineyards through Machine Learning. Data Brief.

[52] Chouhan S.S., Singh U.P., Kaul A., Jain S. A Data Repository of Leaf Images: Practice towards Plant Conservation with Plant Pathology. Proceedings of the 2019 4th International Conference on Information Systems and Computer Networks (ISCON).

[53] Rai N., Mahecha M.V., Christensen A., Quanbeck J., Zhang Y., Howatt K., Ostlie M., Sun X. (2023). Multi-Format Open-Source Weed Image Dataset for Real-Time Weed Identification in Precision Agriculture. Data Brief.

[54] Ahmed S.I., Ibrahim M., Nadim M., Rahman M.M., Shejunti M.M., Jabid T., Ali M.S. (2023). MangoLeafBD: A Comprehensive Image Dataset to Classify Diseased and Healthy Mango Leaves. Data Brief.

[55] Orka N.A., Uddin M.N., Toushique F.M., Hossain M.S. (2023). OLID I: An Open Leaf Image Dataset for Plant Stress Recognition. Front. Plant Sci..

[56] Thapa R., Zhang K., Snavely N., Belongie S., Khan A. (2020). The Plant Pathology Challenge 2020 Data Set to Classify Foliar Disease of Apples. Appl. Plant Sci..

[57] Giselsson T.M., Jørgensen R.N., Jensen P.K., Dyrmann M., Midtiby H.S. (2017). A Public Image Database for Benchmark of Plant Seedling Classification Algorithms. arXiv.

[58] Singh D., Jain N., Jain P., Kayal P., Kumawat S., Batra N. PlantDoc: A Dataset for Visual Plant Disease Detection. Proceedings of the 7th ACM IKDD CoDS and 25th COMAD.

[59] Justina M.J., Thenmozhi M. (2024). SorghumWeedDataset_Classification and SorghumWeedDataset_Segmentation Datasets for Classification, Detection, and Segmentation in Deep Learning. Data Brief.

[60] Guo Z., Goh H.H., Li X., Zhang M., Li Y. (2023). WeedNet-R: A Sugar Beet Field Weed Detection Algorithm Based on Enhanced RetinaNet and Context Semantic Fusion. Front. Plant Sci..

[61] Daphal S., Koli S. (2022). Sugarcane Leaf Disease Dataset. https://data.mendeley.com/datasets/9424skmnrk/1.

[62] Mignoni M.E., Honorato A., Kunst R., Righi R., Massuquetti A. (2022). Soybean Images Dataset for Caterpillar and *Diabrotica speciosa* Pest Detection and Classification. Data Brief.

[63] Alam M.S., Alam M., Tufail M., Khan M.U., Güneş A., Salah B., Nasir F.E., Saleem W., Khan M.T. (2022). TobSet: A New Tobacco Crop and Weeds Image Dataset and Its Utilization for Vision-Based Spraying by Agricultural Robots. Appl. Sci..

[64] Lac L., Keresztes B., Louargant M., Donias M., Da Costa J.P. (2022). An Annotated Image Dataset of Vegetable Crops at an Early Stage of Growth for Proximal Sensing Applications. Data Brief.

[65] Zhang C. (2023). Zhangchuanyin/Weed-Datasets. https://github.com/zhangchuanyin/weed-datasets.

[66] Getachew H. (2021). Wheat Leaf Dataset. https://data.mendeley.com/datasets/wgd66f8n6h/1.

[67] Du Y., Zhang G., Tsang D., Jawed M.K. Deep-CNN Based Robotic Multi-Class under-Canopy Weed Control in Precision Farming. Proceedings of the 2022 International Conference on Robotics and Automation (ICRA).

[68] Güldenring R., Van Evert F.K., Nalpantidis L. (2023). RumexWeeds: A Grassland Dataset for Agricultural Robotics. J. Field Rob..

[69] Hoang Trong V., Yu G.-h., Thanh Vu D., Kim J.-y. (2020). Late Fusion of Multimodal Deep Neural Networks for Weeds Classification. Comput. Electron. Agric..

[70] Olaniyi O.M., Salaudeen M.T., Daniya E., Abdullahi I.M., Folorunso T.A., Bala J.A., Nuhu B.K., Adedigba A.P., Oluwole B.I., Bankole A.O. (2023). Development of Maize Plant Dataset for Intelligent Recognition and Weed Control. Data Brief.

